# Identification of a Novel UT-B Urea Transporter in Human Urothelial Cancer

**DOI:** 10.3389/fphys.2017.00245

**Published:** 2017-04-28

**Authors:** Ruida Hou, Mehrdad Alemozaffar, Baoxue Yang, Jeff M. Sands, Xiangbo Kong, Guangping Chen

**Affiliations:** ^1^Department of Urology, China-Japan Union Hospital, Jilin UniversityChangchun, China; ^2^Department of Physiology, Emory University School of MedicineAtlanta, GA, USA; ^3^Department of Urology, Emory University School of MedicineAtlanta, GA, USA; ^4^Department of Pharmacology, School of Basic Medical Sciences, Peking UniversityBeijing, China; ^5^Renal Division Department of Medicine, Emory University School of MedicineAtlanta, GA, USA

**Keywords:** urothelium, tumor, urea transporter, gene expression, sialylation

## Abstract

The urea transporter UT-B is widely expressed and has been studied in erythrocyte, kidney, brain and intestines. Interestingly, UT-B gene has been found more abundant in bladder than any other tissue. Recently, gene analyses demonstrate that SLC14A1 (UT-B) gene mutations are associated with bladder cancer, suggesting that urea transporter UT-B may play an important role in bladder carcinogenesis. In this study, we examined UT-B expression in bladder cancer with human primary bladder cancer tissues and cancer derived cell lines. Human UT-B has two isoforms. We found that normal bladder expresses long form of UT-B2 but was lost in 8 of 24 (33%) or significantly downregulated in 16 of 24 (67%) of primary bladder cancer patients. In contrast, the short form of UT-B1 lacking exon 3 was detected in 20 bladder cancer samples. Surprisingly, a 24-nt in-frame deletion in exon 4 in UT-B1 (UT-B1Δ24) was identified in 11 of 20 (55%) bladder tumors. This deletion caused a functional defect of UT-B1. Immunohistochemistry revealed that UT-B protein levels were significantly decreased in bladder cancers. Western blot analysis showed a weak UT-B band of 40 kDa in some tumors, consistent with UT-B1 gene expression detected by RT-PCR. Interestingly, bladder cancer associate UT-B1Δ24 was barely sialylated, reflecting impaired glycosylation of UT-B1 in bladder tumors. In conclusion, SLC14A1 gene and UT-B protein expression are significantly changed in bladder cancers. The aberrant UT-B expression may promote bladder cancer development or facilitate carcinogenesis induced by other carcinogens.

## Introduction

Urea is the major end product of nitrogen metabolism and is excreted by the kidney. Due to its small molecular size (~60 Da) and water solubility, urea has been considered to be freely permeable across the cell membrane for over 30 years (Sands, 2003a, 2007). In fact, as a highly polar molecule, urea permeability across lipid bilayers is very low. Urea transport across the cell membrane is mediated by a facilitated urea transporter (Klein et al., 2011; Li et al., 2012a). In mammals, there are two types of urea transporters, UT-A and UT-B, encoded by the solute carrier family SLC14A2 and SLC14A1 genes, respectively. These genes share a high degree of homology and are aligned in tandem at chromosome 18q12.3 in humans. UT-A urea transporters are mainly expressed in kidney epithelial cells while UT-B urea transporters demonstrate a broader tissue distribution including bladder (Timmer et al., 2001; Spector et al., 2004, 2007; Dong et al., 2013). UT-B also serves as a determinant antigen of the Kidd blood group on erythrocytes (Sands, 2003b; Shayakul et al., 2013).

Compared to the kidney UT-A urea transporter, UT-B is under-investigated. Two types of human UT-B are reported. UT-B1 (Genbank NM_001146036) was first cloned from human bone marrow (Olives et al., 1994) and expressed in multiple tissues including brain, heart, kidney, bladder, prostate, etc. (Timmer et al., 2001; Yang et al., 2002). UT-B2, however, was first identified from bovine rumen as bovine UT-B2 (bUT-B2), with an additional 56-amino acid spliced into the N-terminal of the original bovine UT-B1 sequence (Stewart et al., 2005). Both UT-B2 mRNA and protein have been detected in cow rumen (Stewart et al., 2005). The UT-B2 mRNA was initially reported in humans in the caudate nucleus (Genbank NM_001146037) (Stewart, 2011), and recently also in the bladder (Walpole et al., 2014).

In the kidney, urea is reabsorbed by UT-A to establish the medullary osmolarity gradient enabling the concentration of urine (Fenton et al., 2004; Sands, 2007). However, extra-renal urea accumulation in tissues is usually detrimental to cells. High urea exposure can affect cells in many ways, such as destroying protein hydrophobic bonds and causing protein carbamylation (Zou et al., 1998), inducing oxidative stress, DNA damage and apoptosis (Michea et al., 2000; Zhang et al., 2004; Dong et al., 2013). Therefore, urea transporters (mainly UT-B) in non-renal tissues function as scavengers that prevent intracellular urea aggregation and intoxication (Guo et al., 2007; Meng et al., 2009; Li et al., 2012b). Studies in UT-B knockout mice demonstrate that absence of UT-B results in notable urea accumulation, abnormal morphology in the hippocampus and depression-like behavior (Li et al., 2012b). Deletion of the SLC14A1 gene causes urea accumulation in the testis, increases testicular weight and results in early maturation of the male reproductive system (Guo et al., 2007). Dong et al. reported that the urothelium urea concentration is 9 times higher in UT-B knockout mice. This is accompanied by increased cell DNA damage, apoptosis and malfunction of arginine metabolism (Dong et al., 2013). In addition, UT-B suppresses tumor growth; overexpression of UT-B inhibits colony formation of lung cancer cell lines transfected with the SLC14A1 genes (Frullanti et al., 2012).

Bladder urothelium expresses UT-B but not UT-A (Yang et al., 2002; Dong et al., 2013; Walpole et al., 2014). This was confirmed recently by an RNA-sequencing analysis showing high expression of SLC14A1 and the absence of SLC14A2 in bladder tissue (Koutros et al., 2013). Using multiple mouse tissues, Yang et al. reported in 2002 that the bladder expresses the most UT-B mRNA *in vivo* (Yang et al., 2002). However, the physiological significance of bladder UT-B is unknown. Recently, two large-scale genome wide association studies (GWAS) of urothelial bladder cancer by two separate groups discovered that mutations of the SLC14A1 gene are linked to bladder carcinogenesis in humans (Garcia-Closas et al., 2011; Rafnar et al., 2011). This suggests that loss of UT-B function may play an important role in suppressing bladder cancer. Considering that the bladder is a reservoir of urine and the urothelial cells are constantly in contact with a high urea concentration, it is not very surprising that the bladder needs more urea transporter UT-B than any other organ (Yang et al., 2002).

In this study, we examined UT-B gene and protein expression in human bladder cancer samples. We found that normal bladder expresses UT-B2. However in bladder cancer, UT-B2 gene expression was suppressed. Instead, bladder cancer expresses the short form of the UT-B1 gene: 11 of 20 (55%) of UT-B1 transcripts are tumor specific UT-B1 with a 24-nt in-frame deletion. We also examined UT-B protein expression and found decreased UT-B expression associated with tumor malignancy.

## Methods

### Cell lines and culture

Normal human urothelial cells (NHU) were kindly provided by Dr. Jennifer Southgate (University of York, UK) and cultured as previously described (Wezel et al., 2013). Primary bladder cancer-derived cell lines including T24 cells (human muscle invasive bladder cancer cell line HTB-4) and 5637 cells (human non-muscle invasive bladder cancer cell line HTB-9) were purchased from the American Type Culture Collection (ATCC, Manassas, VA) and cultured in DMEM (GIBCO, Waltham, MA) supplied with 10% FBS.

### Bladder tumor specimen collection

Fresh bladder cancer samples were taken immediately after surgery by the Emory University Hospital Department of Urology and stored at −80°C for further use. All tissue samples were obtained from patients who consented through an IRB approved protocol (IRB: 00055316) at Emory University to have excess pathological specimens for research purposes.

### RNA extraction, cDNA synthesis, and PCR amplification

**Figure 1:**
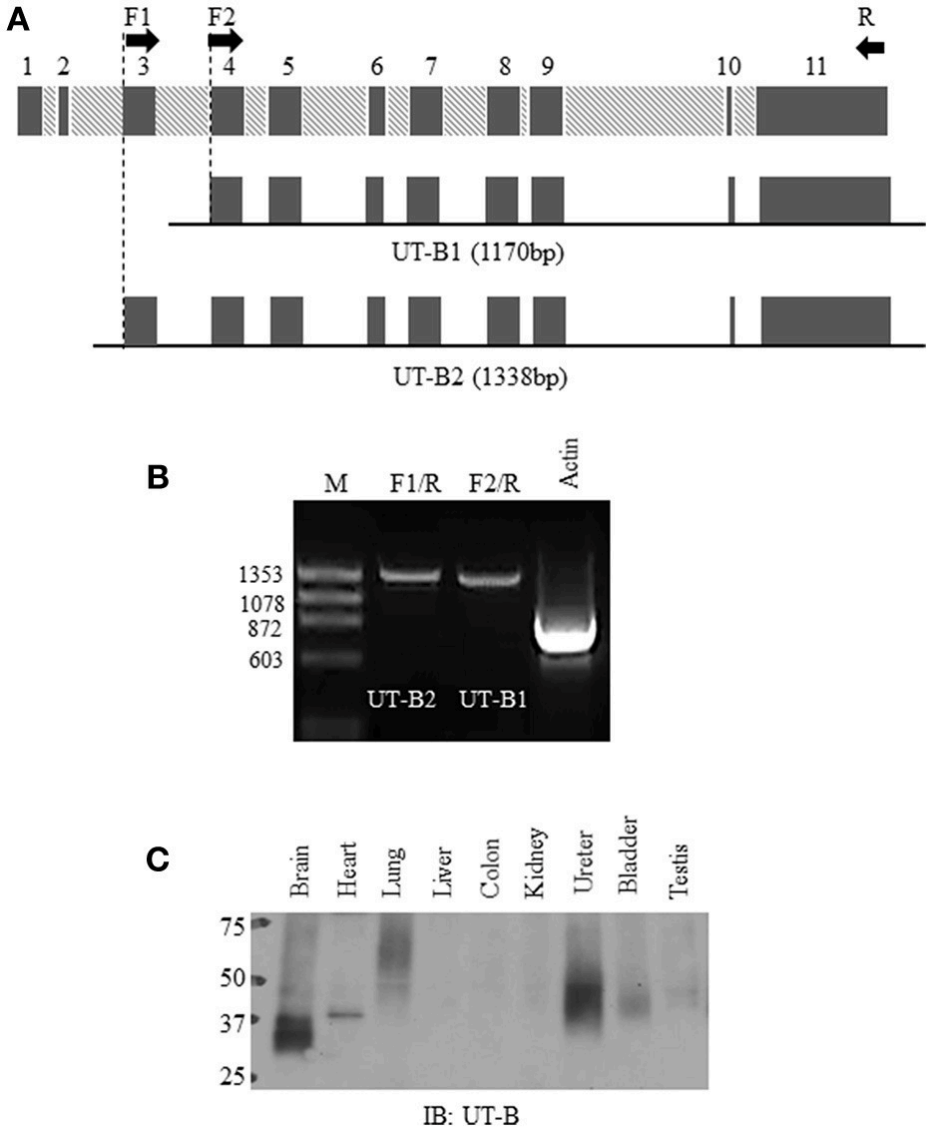
**Normal bladder urothelium expresses UT-B2 isoform. (A)** Schematic diagram of human SLC14A1 gene and two pairs of primers for RT-PCR. **(B)** RT-PCR. PCR amplification of UT-B2 and UT-B1 from normal bladder tissue cDNA. GAPDH was used as an internal control. **(C)** Western blot analysis of UT-B protein expression in human tissues. The pre-made human tissue blot was purchased from Protein Biotechnologies and probed with UT-B antibody.

Total RNA was extracted from tissues using TRIzol reagents (Invitrogen, Carlsbad, CA). The RNA integrity was evaluated by RNA electrophoresis and an Agilent Bioanalyzer 2100. Three microgram of total RNA was used for cDNA synthesis. Reverse transcription (RT) was carried out in a 20 μl reaction using SuperScript First-Strand Synthesis System for RT-PCR (Invitrogen 11904-018). One microliter of cDNA was used for PCR using Advantage 2 Polymerase Mix (Clontech 639201100, Mountain View, CA). Two pairs of UT-B primers were designed according to the human UT-B gene (NM_001128588) sequence and used for amplification of UT-B2 or UT-B1 (Figure 1A). All amplified products were ligated to TOPO TA vector (Invitrogen K4500-01) and submitted for DNA sequencing.

### Quantitative real-time PCR assays

Quantitative real-time PCR (qPCR) was performed as described before (Chen et al., 2010; Qian et al., 2015). Gene specific primers for UT-B1, UT-B2, and GAPDH were designed to generate amplicons of length 100–200 nucleotides by using the Invitrogen Primer program. The sequences of PCR primers for real-time PCR used were 5′-ccagtgggagttggtcagat-3′ (sense) and 5′-gttgaaaccccagagtccaa-3′ for UT-B1, 5′-aggacccttttggaactaaagc-3′ and 5′-gggctgtccactctaaccatag-3′ for UT-B2, 5′-cgagatccctccaaaatcaa-3′ and 5′-ttcacacccatgacgaacat-3′ for GAPDH. Prior to quantitative PCR, a single amplified product of the predicted size was verified by regular PCR and DNA sequencing. Real-time PCR was carried out using the Bio-Rad iCycler Real-Time Detection System with a two-step protocol (3 min at 95°C; followed by 40 cycles of 10 s at 95°C and 45 s at 61°C). Fluorescence of the amplificates was detected with the Brilliant III Ultra-Fast SYBR Green QPCR Master Mix (Agilent). The cycle threshold number (Ct) for each sample was determined at a constant fluorescence threshold by iCycler software 3.0 (Bio-Rad). The experiments were repeated at least twice and all reactions were performed in triplicate. The gene expression data were analyzed using the 2^ΔCt^ formula, which ΔCt = Ct _UT-B_ - Ct _GAPDH_. Significance was determined using a two-sample, two tailed Student's *t*-test.

### Plasmid construction

The human UT-B2 and UT-B1 cDNAs were PCR amplified from normal human bladder cDNA (BioChain, C1234010, Newark, CA). UT-B1Δ24 cDNA was PCR amplified from human bladder tumor cDNA (BioChain, C1235010). The UT-B cDNAs were constructed into the mammalian expression vector pcDNA3 or into an oocyte expression vector pGH19. All constructs were verified by DNA Sequencing (Beckman Counter Genomics, Beverly, MA).

### Peptide-N-glycosidase F (PNGase F) treatment

Human UT-B2, UT-B1, and UT-B1Δ24 in pcDNA3 were transfected into HEK293 (Stratagene, San Diego, CA) cells using Lipofectamine2000 (Invitrogen) following the manufacturer‘s instructions. After 48 h, cells were solubilized in RIPA buffer (150 mM NaCl, 10 mM Tris·HCl, pH 7.5, 1 mM EDTA, 1%, Triton X-100, 1% sodium deoxycholate and 0.1% SDS). Lysates were first denatured with glycoprotein denaturing buffer at 100°C for 10 min, then 10% NP-40 buffer and G7 reaction buffer were added, and incubated with or without 1 μl PNGase F (500 units/μl, New England Biolabs, Ipswich, MA) at 37°C for 2 h. UT-B proteins were examined by western blot analysis.

### Protein extraction and western blot

Bladder cancer tissues were lysed in ice-cold RIPA buffer. After centrifugation at 10,000 rpm at 4°C for 10 min, supernatants were collected. The protein concentration was determined by a Bradford method with the Protein Assay reagent (Bio-Rad Laboratories, Hercules, CA). Proteins (20~50 μg/lane) were size separated by SDS-PAGE and electroblotted to the nitrocellulose membrane. The membranes were routinely processed by blocking with 5% milk/PBS, incubation overnight with primary rabbit polyclonal anti-UT-B antibody (Doran et al., 2006) and incubation for 1 h with horseradish peroxidase-conjugated secondary antibody. Immunoreacting proteins were detected using an Enhanced Chemiluminescence (ECL) Kit (Amersham, Pittsburgh, PA).

### Immunohistochemistry

Bladder cancer tissue microarray T122, BL803, and BL244 were purchased from US Biomax Inc. (Rockville, MD). Bladder cancer tissue array TMA-2205 was from Protein Biotechnologies (Ramona, CA). Bladder cancer tissue array BLC241 was from Pantomics Inc. (Richmond, CA). The tissue arrays were de-paraffinized in xylene and rehydrated. For antigen retrieval, the tissue array slides were immersed in 0.01 M citrate buffer for 3 min in a microwave oven. Endogenous peroxidase was blocked with 0.3% hydrogen peroxidase in methanol for 20 min. After blocking with 1% BSA at room temperature for 20 min, the tissues were incubated in a humidified chamber with UT-B antibody (1:400) at 4°C overnight. After a PBS wash, tissues were incubated with biotinylated anti-rabbit antibody, followed by avidin-biotin peroxidase complex (ABC) (Vector laboratories, Burlingame, CA) at room temperature for 1 h. Antibody localization was detected with diaminobenzidine (DAB) as a chromogen substrate. Nuclei were counterstained with hematoxylin. The UT-B protein expression was examined under a microscope and semi-quantified as 0 ~ 4 scale: 0 = no appreciable staining or faint staining intensity in < 10% of tumor cells, 1 = faint staining in >10% of tumor cells, 2 = readily appreciable brown staining, 3 = dark brown staining, or 4 = very strong staining.

### Lipid raft isolation and lectin pulldown assay

Lipid raft isolation was performed as previously described (Chen et al., 2011). Briefly, fresh bladder cancer tissues or UT-B transfected HEK293 cells were homogenized in ice-cold 0.5% Brij96/TNEV buffer (10 mM Tris·HCl pH 7.5, 150 mM NaCl, 5 mM EDTA and 2 mM Na_3_VO_4_) on ice for 30 min. Supernatants were loaded in a 5–40% discontinuous sucrose gradient. After centrifugation at 34,000 rpm for 16 h, an equal amount of each fraction (approximately 400 μl) was collected from the top to the bottom. UT-B in the lipid rafts were examined by western blot.

For lectin pulldown assay, equal amounts of lipid raft membrane fractions were incubated with agarose-conjugated lectins at 4°C overnight. Lectin precipitated UT-B were detected by Western blot with anti-UT-B antibody. Agarose-bound concanavalin A (ConA), galanthus nivalis lectin (GNL), wheat germ agglutinin (WGA) and Sambucus nigra lectin (SNA) were purchased from Vector Laboratories (Burlingame, CA).

### Water permeability experiments in oocytes

Xenopus laevis oocytes were prepared and maintained in OR3 medium as previously described (Chen et al., 2011). Capped UT-B cRNAs were transcribed from linearized pGH19-UT-B using the mMESSAGE mMACHINE T7 Ultra Kit (Ambion). Two ng of UT-B cRNAs were microinjected into oocytes. Three days later, functional study of oocyte water permeability (Chen et al., 2015) was measured by placing oocytes in a low osmotic solution (one volume of ND96 mixed with one volume of water). The cell rupture time in the hypo-osmolarity solution was counted by visual inspection using a microscope.

### Statistical analysis

The gene expression data collected from qPCR were analyzed using the 2^ΔCt^ formula, which Δ Ct = Ct _UT-B_ - C-t_GAPDH_. Significance was determined using a two-sample, two tailed Student's t-test. Western blot densities were analyzed with ImageJ. Data collected from Western blot, immunohistochemistry and water permeability experiment in oocytes were performed by one-way analysis of variance (ANOVA) followed by Tukey HSD tests. And values were expressed as means ± SD.

## Results

### Normal human bladder urothelium expresses UT-B2

Bladder urothelium expresses urea transporter UT-B (Li et al., 2014; Walpole et al., 2014). However, it remains unclear which forms of UT-B are expressed in human bladder. Figure 1A illustrates human SLC14A1 (UT-B) gene with 11 exons. UT-B2 is the large form of UT-B containing exon 3. We designed two pairs of UT-B primers to amplify UT-B2 and UT-B1 from normal human bladder tissue cDNAs purchased from BioChain. Primer location and sequences are shown in Table 1. Clearly, UT-B2 (1,338 bp) and UT-B1 (1,170 bp) were successfully amplified from human bladder (Figure 1B). The amplified products were verified by DNA sequencing. We then examined UT-B protein expression using a pre-made human tissue blot (Protein biotechnologies). A band around 40–48 kDa was identified in bladder (Figure 1C), suggesting that under normal condition, bladder mainly expresses UT-B2. UT-B signals were also detected in human brain, lung, kidney, ureter and testis.

**Table 1:** **Primers for amplification of human UT-B transcripts**.

**Name**	**Location and description**	**Sequence**
F1	Exon 3, Forward primer for hUT-B2	5′-CGGGATCCGGATGAATGGACGGTCTTTGATTG-3′
F2	Exon 4, Forward primer for hUT-B1	5′-CGGGATCCATGGAGGACAGCCCCACTATGGTTAG-3′
R	Exon 11, Reverse primer	5′-GCTCTAGATTCTCACAAAGGGCTTTCCACCATTC-3′

### Bladder cancer switches to express UT-B1 gene

**Figure 2:**
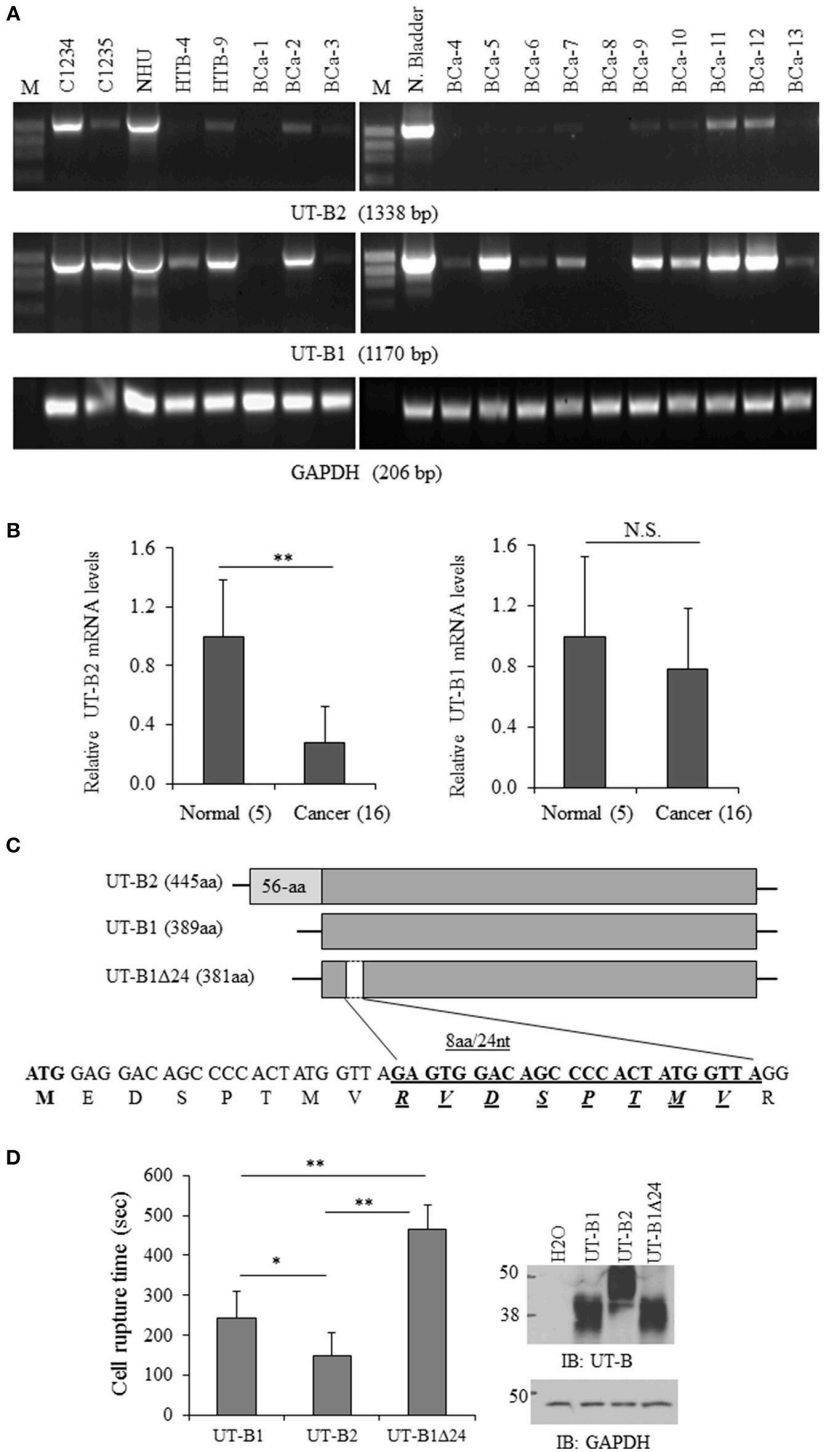
**Analysis of UT-B gene expression from bladder cancer patients. (A)** Representative RT-PCR amplification of UT-B by two pairs of primers. BCa-1 to Bca-13 are fresh bladder cancer tissues (from total *n* = 24) collected from patients and used for RT-PCR (C1234: normal bladder cDNA, C1235: bladder cancer cDNA, NHU: normal human urothelial cell line, HTB-4 and HTB-9: bladder cancer derived cell lines). **(B)** Quantification of UT-B gene expression by real-time PCR. UT-B gene expression was assessed in normal and cancerous tissues by real-time PCR using gene-specific primers and SYBR Green detection. Gene expression was determined using the 2^ΔCt^ method with GAPDH as control gene. Relative mRNA levels of UT-B in cancer (*n* = 16) were compared to the normal bladder tissues (*n* = 5), where the expression was set to 1.0 (mean ± SD, compared to control ^**^P < 0.01; NS, no significance). **(C)** Schematic diagram of UT-B2, UT-B1 and UT-B1 Δ24 and the location of deletion in UT-B1. **(D)** Functional study of UT-B activity. UT-B cRNAs (2ng/cell) were injected into oocytes. Three days later, UT-B activity was assessed by water permeability assay by placing cells in hypo-osmolarity solution. The cell rupture time was counted (*n* = 7–10 cells/group) and the significance was determined by ANOVA (^*^p < 0.05, ^**^p < 0.01). UT-B protein expression was examined by western blot with UT-B antibody (*n* = 10 cells/group).

Next, we examined UT-B gene expression in bladder cancer tissues and bladder cancer cell lines. In total 24 fresh bladder cancer tissue samples were collected. The normal human bladder cDNA C1234 and bladder tumor cDNA C1235 from BioChain, normal bladder cell NHU and two bladder cancer derived cell lines (HTB-4 and HTB-9) were used as controls. Using the primers F1/R, UT-B2 (1,338 bp) was amplified in NHU cell and normal human bladder samples (Figure 2A). UT-B2 was dramatically suppressed in tumor and tumor cells (Figure 2A upper panel). We then investigated UT-B1 transcript expression by primers F2/R in bladder cancer and cancer cells. Interestingly, 16 out of 24 bladder cancer expressed UT-B1 (1,170 bp) which lacks exon 3 (Figure 2A middle panel). This indicates that bladder cancer cells somehow switch to express UT-B1 instead of UT-B2. GAPDH (Figure 2A lower panel) was used as an internal control. The changes of UT-B1 and UT-B2 mRNA were validated by real-time PCR in 16 bladder cancers and 5 normal human bladder tissues. GAPDH expression was used as reference gene for normalization. Figure 2B displays relative quantity of UT-B mRNA in tumor compared to normal bladder tissues. The mRNA levels of UT-B2 were significantly down-regulated in tumor compared to normal bladder (*p* < 0.01). The large SD reflected the heterogeneity of the patient samples individual gene expression levels.

### Discovery of a 24-nt deletion in UT-B1 gene only from bladder cancer patients

All PCR products of UT-B1 from bladder cancer patients were purified and sent for DNA sequencing. Surprisingly, in 11 of 20 (55%) samples, gene sequencing showed a 24-nucleotide sequence missing in exon 4 that corresponded to a deletion of 8 amino acids. The 24-nt in-frame deletion was not found in normal bladder samples (commercially purchased) and normal bladder NHU cells. Figure 2C illustratesUT-B2, UT-B1, UT-B1Δ24, and the position of 24-nt deletion localized in UT-B1. The deletion starts at the 26th nucleotide downstream of the translation initiation site in UT-B1, and extends over 24 nucleotides. Therefore, bladder cancer expresses tumor specific UT-B1 with a 24-nt deletion (UT-B1Δ24).

UT-B also transports water (Yang and Verkman, 1998). We then evaluated the functional difference of UT-B1, UT-B2, and UT-B1Δ24 by water permeability experiments in oocytes as described before (Chen et al., 2015). The cell rupture time of UT-B1Δ24 in hypo-osmolarity solution was significantly longer than that of UT-B1, reflecting decreased functional activity. The cell rupture time of UT-B2 was shorter than that of UT-B1 (Figure 2D). The average cell rupture time for the water-injected control oocytes is around 5,000 s (data not shown).

### Decreased UT-B1 protein expression in human bladder cancer

**Figure 3:**
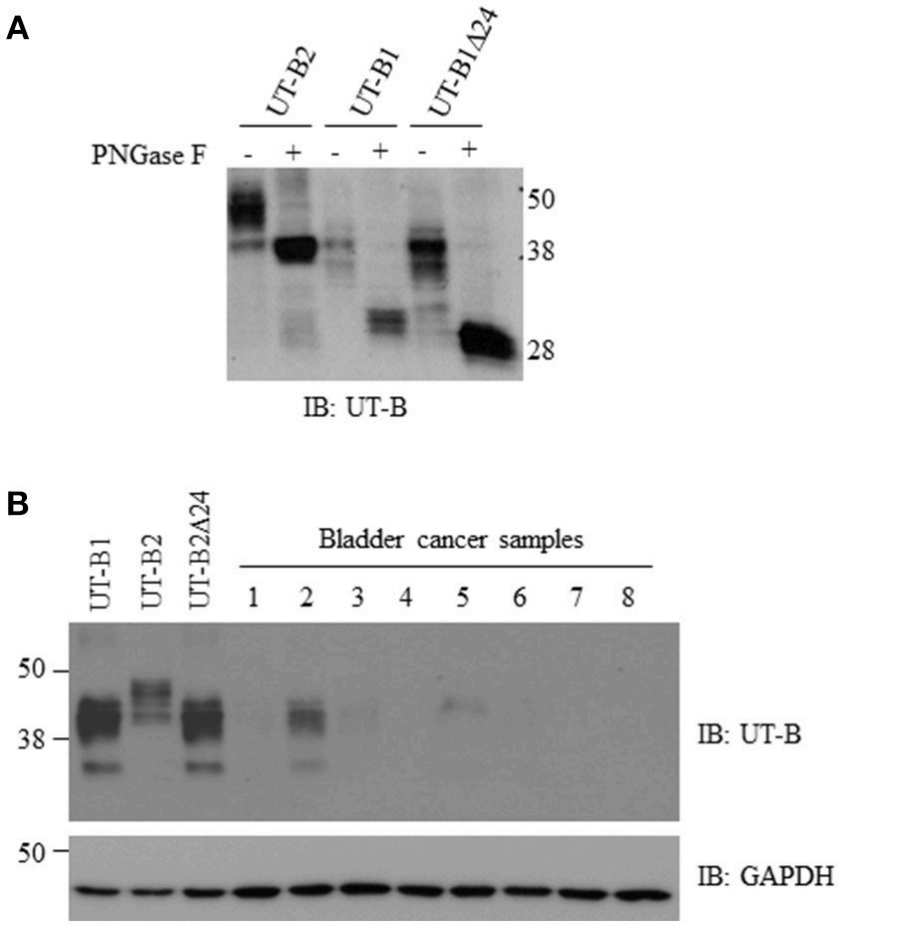
**Glycosylation analysis of UT-B2, UT-B1, and UT-B1Δ24 and western blot analysis of UT-B protein expression from bladder cancer patients. (A)** PNGase F treatment. The cell lysates of HEK293 cell transfected with UT-B2, UT-B1, and UT-B1Δ24 were treated with or without PNGase F at 37°C for 2 h. **(B)** Fresh bladder cancer tissues were lysed in RIPA buffer. Supernatants were collected. UT-B protein expression was examined by western blot with UT-B antibody. Recombinant UT-B1, UT-B2, and UT-B1Δ24 expressed in HEK293 cells were used as controls. The same membrane was stripped and re-probed with GAPDH antibody.

We cloned human UT-B2, UT-B1, and UT-B1Δ24 cDNA and transfected them into HEK293 cells. The recombinant protein sizes and glycosylation states were examined (Figure 3A). Western blot analysis showed ~40 and ~48 kDa for UT-B2. After de-glycosylation enzyme PNGase F treatment, the two bands were collapsed to ~38 kDa, which is consistent with the previous report (Walpole et al., 2014). UT-B1 showed two bands at ~ 35 and ~40 kDa, and the deglycosylated form was ~30 kDa. The size of the two glycosylated forms of UT-B1Δ24 was slightly lower than that of UT-B1. After PNGase F treatment, UT-B1Δ24 showed a single band at ~28 kDa.

We collected fresh tumor samples and examined UT-B protein expression in bladder cancer. Most bladder cancer did not express or expressed a low level of UT-B (Figure 3B). The size is close to UT-B1, indicating that bladder cancer expresses UT-B1 but not UT-B2 isoform. This is consistent with results of RT-PCR in Figure 2A.

### Histological analysis of UT-B protein expression in human bladder cancer

**Figure 4:**
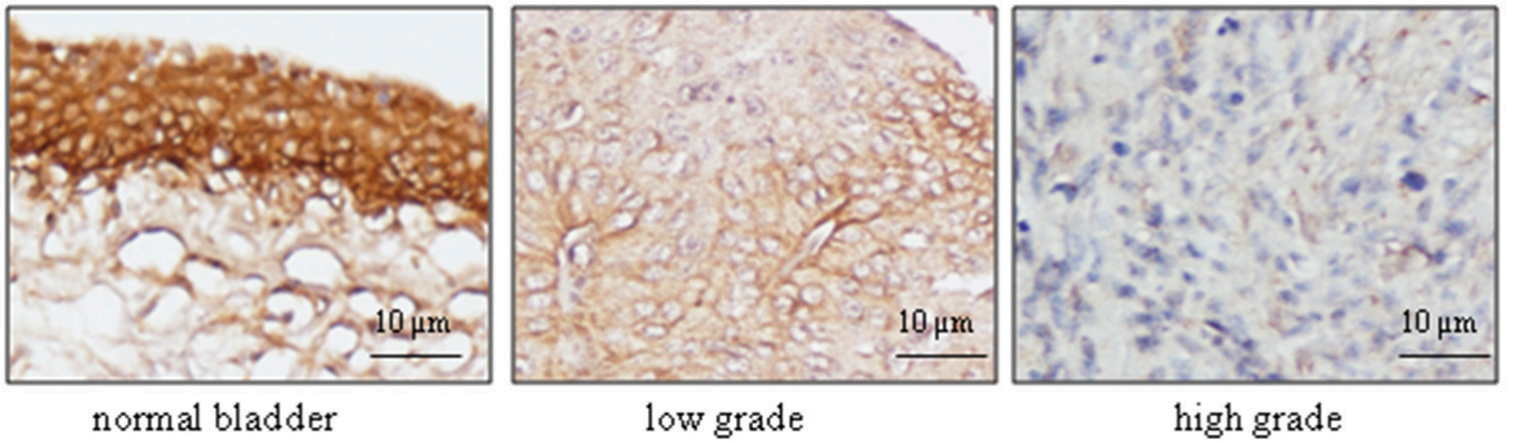
**Immunohistochemical analysis of UT-B expression with bladder cancer tissues**. Human bladder cancer tissue arrays paired with normal tissues were processed for immunostaining with UT-B antibody (1:400), followed by biotinylated anti-rabbit antibody, and ABC (Vector laboratories). Color was developed by DAB. The nuclei were counterstained with hematoxylin. Representative pictures of UT-B staining in bladder cancer are shown from total 63 cases (*n* = 73) (magnification: x200).

To further confirm the finding by western blot showing a decreased level of UT-B in bladder cancer, immunohistochemistry was employed to evaluate UT-B protein expression in situ with human bladder cancer tissues. Six different tissue microarrays of human bladder tumors paired with normal tissues were examined. Excluding adenocarcinoma, 73 cases were patients with urothelial carcinoma. As shown in Figure 4, UT-B protein was detected throughout the urothelium in normal bladder tissues and was decreased in bladder cancer. UT-B protein staining was semi-quantified on a scale of 0~4. The average score of UT-B protein staining was 3.67 ± 0.41 (mean ± SD) in normal bladder urothelium, 1.61 ± 0.53 in low grade urothelial carcinoma, and 0.52 ± 0.43 in high grade urothelial carcinoma (Table 2), showing a strong association of UT-B protein expression with tumor malignancy.

**Table 2:** **Semi-quantification of UT-B expression in bladder cancer**.

**Grade**	**N**	**IHC score**
Control	73	3.67 ± 0.41
Low grade	45	1.61 ± 0.53^a^
High grade	28	0.52 ± 0.43^a^^,^^b^

a Compared to control p < 0.01;

b *Compared to low grade p < 0.01*.

### Hypo-sialylation modification of UT-B1 from bladder cancer

**Figure 5:**
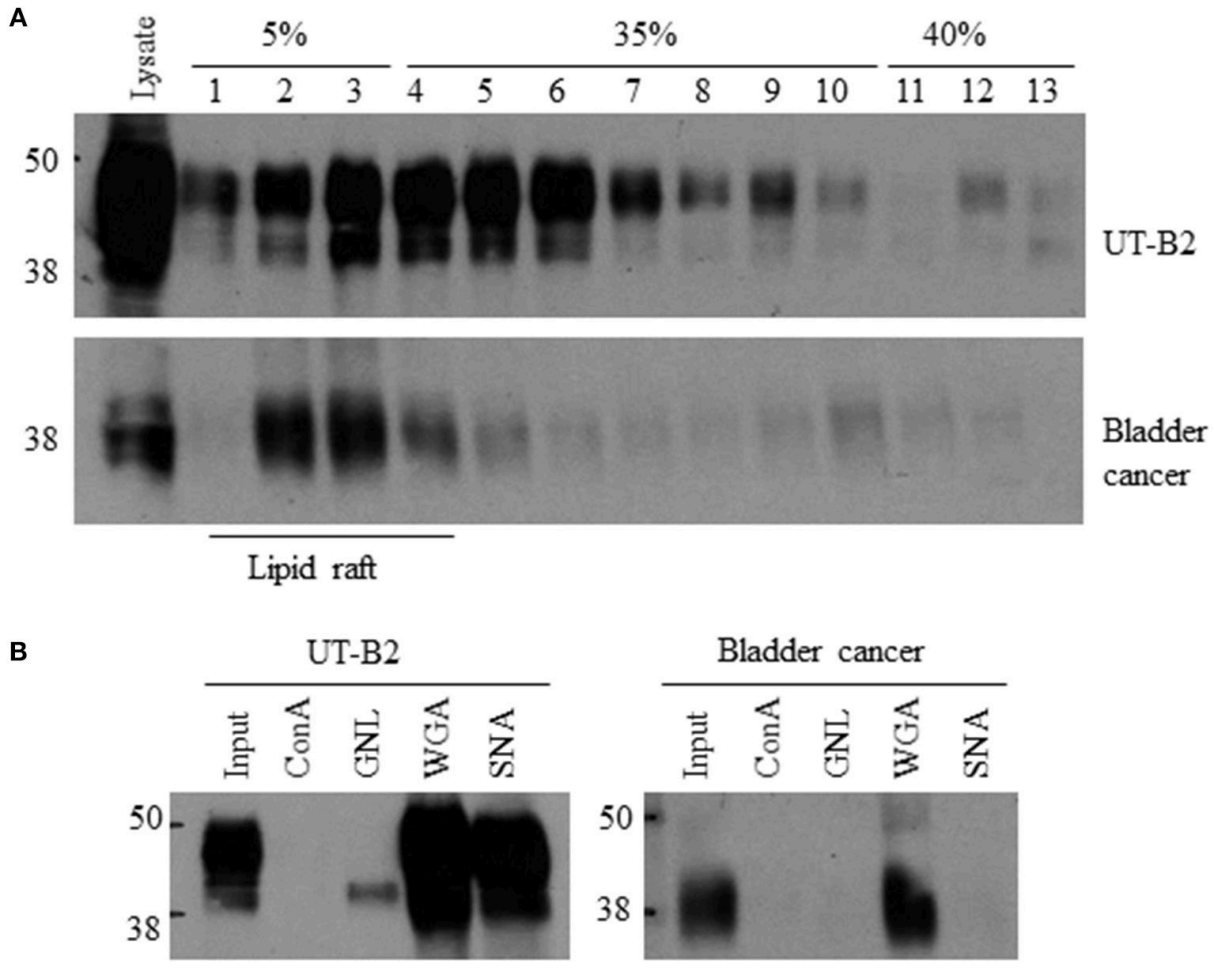
**Lipid raft distribution and glycan structure analysis of bladder cancer UT-B. (A)** UT-B cell membrane distribution. Fresh bladder cancer tissues were lysed in 0.5% Brij96/TNEV. Supernatants were loaded in a 5–40% sucrose gradient for ultracentrifugation. Fractions were collected and UT-B was examined by western blot. UT-B2 expressed in HEK293 cells was used as a control. **(B)** Lectin pulldown assay. Lipid raft fractions 2–5 were collected from the samples above and incubated with different lectins. Lectin precipitated UT-B was examined by western blot. Representative figures are shown from 5 independent experiments. ConA, Concanavalin A; GNL, Galanthus nivalis lectin; WGA, Wheat germ agglutinin; SNA, Sambucus nigra lectin.

Glycosylation is a key post-translational modification that can affect the structure and function of glycoproteins. As shown in Figure 3A, UT-B is a highly glycosylated protein. A single N-glycosylation site was identified at Asn 211 in the UT-B protein (Shayakul et al., 2013). We previously reported that cell membrane UT-A1 and UT-A3 are localized in lipid raft microdomains and glycosylation facilitates UT-A1 lipid raft distribution in lipid rafts (Chen et al., 2011; Su et al., 2012). We examined bladder cancer UT-B in cell membrane localization using fresh bladder cancer tissues from patients. UT-B2 expressed in HEK293 cells was used as a control. As shown in Figure 5A, UT-B1from bladder cancer was expressed in lipid rafts in the cell membrane, although the total UT-B1 protein level was low.

We then collected lipid raft fractions and profiled the glycan structure of UT-B1 from bladder cancer patients by lectin pulldown. Lectin is a group of proteins specifically recognizing carbohydrates. As shown in Figure 5B, the 40–48 kDa UT-B2 band was mainly bound by WGA and SNA, which recognize N-acetylglucosamine and α-2,6 sialic acid, respectively (Chen et al., 2011; Su et al., 2012). The lower band of UT-B2 contained mannose, which was pulled down by GNL. Interestingly, the UT-B1 from bladder cancer contains N-acetylglucosamine pulled down by WGA. However, bladder cancer UT-B1 was not pulled down by SNA, indicating an impaired glycan sialylation of UT-B1 from bladder cancer.

## Discussion

Bladder cancer is one of the most common cancers in the United States with 74,000 estimated cases and 16,000 estimated deaths in 2015 (Siegel et al., 2015). Despite significant progress in the cancer research field overall, little has been achieved in bladder cancer in the same time and the molecular mechanism of bladder tumorigenesis and cancer progression remains poorly understood. In this study, we found that the SLC14A1 gene, which encodes urea transporter UT-B and mediates urea transport across the cell membrane, was suppressed or aberrantly expressed in bladder cancer. We, for the first time, identified a tumor specific UT-B1Δ24 with a 24-nt in-frame deletion in exon 4 from bladder cancer patients. Consistently, UT-B protein was significantly decreased in bladder cancer in our study and others (Li et al., 2014). Thus, a significant alteration of the UT-B gene/protein in bladder tumors supports the potential tumor suppressive role of UT-B in bladder cancer carcinogenesis.

The bladder is a unique urine storage organ dealing with highly concentrated urea. The “urogenous contact hypothesis” proposed by Braver (Braver et al., 1987) in 1987 is still prevalent in the etiology of bladder cancer. Environmental exposures, particularly tobacco smoking, are important risk factors in bladder cancer. However, one important factor we may have ignored in bladder carcinogenesis is urea. High intracellular urea concentration damages cells in many ways. The urea concentration in urine is 20–100 times higher than in blood in humans (Yang and Bankir, 2005; Spector et al., 2007). Bladder urothelium is inevitably and constantly insulted by a higher concentration of urea than any other tissue/organ. This could be the reason why urothelium expresses the most abundant UT-B when compared to any other tissue (Yang et al., 2002). It is not very surprising that genomic analysis from two groups discovered UT-B gene mutations as bladder cancer susceptibility genes (Garcia-Closas et al., 2011; Rafnar et al., 2011).

UT-B has two isoforms, UT-B1 with 389 aa and UT-B2 with 445 aa. UT-B2 is the longer form. UT-B1, which lacks exon 3, has been identified in multiple tissues. UT-B2 with an extra 56 aa in the N-terminus is only reported in the human caudate nucleus (Stewart, 2011), mouse thymus (Yang et al., 2002), and bovine rumen (Tickle et al., 2009). In this study, we found that normal bladder urothelium expresses UT-B2. We also found that, for an unknown reason, UT-B2 expression was suppressed in bladder tumors. Instead, tumor cells switched to expression of the short form of UT-B1.

Gene deletion and mutation are frequently involved in the development of human urothelial carcinoma, such as the deletion in chromosome 9 (Schulz, 2006; Knowles, 2008), point mutations of the fibroblast growth factor receptor-3 (FGFR3) (Iyer and Milowsky, 2013; Pandith et al., 2013), and alterations in tumor suppressor gene TP53 and RB1 (Mitra et al., 2006) and FHIT (Baffa et al., 2000). In this study, we found a 24-nt in-frame deletion in UT-B1 (UT-B1Δ24) in a high incidence (55%) from bladder cancer patients. This deletion has never been reported before. The cause of this deletion currently is unknown. Notably, the SLC14A1 (18q12.1-18q21.1) gene happens to be localized at one of the common fragile sites on chromosome 18(18q12.2, 18q21.3) (Durkin and Glover, 2007). Interestingly, the in-frame deletion starts from nt 26 and is not in a triple reading frame. However, this does not interrupt the blueprint of protein translation except for an 8-aa missing. The in-frame deletion has been reported in multi gene mutations. Chloride channel CFTR ΔF508 has a 3-bp in-frame deletion in exon 10 and causes a single amino acid deletion at F508. CFTR ΔF508 is the most common cystic fibrosis causing genetic mutation (Kalin et al., 1999; Lukacs and Verkman, 2012). Greater than 90% of EGFR tyrosine kinase domain mutations are short in-frame deletions in exon 19 with different deletion sizes (9, 12, 15, 18, and 24 bp; Bethune et al., 2010; Brevet et al., 2010). Evaluation of EGFR mutation has been employed to predict lung adenocarcinoma response to EGFR kinase inhibitors in clinic (Brevet et al., 2010). It is also interesting whether assessment of UT-BΔ24 could guide the selection of bladder cancer treatment in the future.

Sialylation modification of glycoproteins plays crucial roles in many biological processes of human health and disease (Park et al., 2012). The alteration of cell membrane protein sialylation is often involved in the development of cancer and tumor metastasis (Pinho and Reis, 2015; Vajaria et al., 2015). Using specific lectin SNA, we found that UT-B1 from bladder cancer has defective alpha-2,6-sialylation. This is consistent with a study by Antony et al. showing that tumor-specific DNA methylation of the ST6GAL1 promoter occurs in human bladder cancer (Antony et al., 2014). The ST6GAL1 gene encodes the alpha-2,6-sialyltransferase, which catalyzes glycan alpha-2,6 sialylation. Presumably, the impaired sialylation of UT-B might be due to inactivation of ST6GalI in bladder cancer. Therefore, in bladder cancer, not only is UT-B protein decreased, but UT-B sialylation is also affected.

Additionally, a unique feature of bladder cancer is that up to 70% of these patients will experience relapse within 5 years after the first standard transurethral resection of the bladder tumor (TURBT), the highest rate of recurrence of any cancer (Al-Sukhun and Hussain, 2003; Chamie et al., 2013). Considering that the bladder urothelium is bathed in fluid with a high urea concentration, loss of UT-B protection exposes the urothelium to constant attack by urea. Therefore, dysfunction of UT-B may contribute to high rates of bladder cancer recurrence.

In summary, the significant amount of UT-B expressed in bladder (Yang et al., 2002) suggests that UT-B is important for the bladder cells to balance intracellular urea and protect them from damage caused by the high urine urea concentration. In this study, we found that the normal UT-B2 gene is suppressed in bladder cancer. Bladder cancer cells aberrantly express UT-B1 with a 24-nt deletion. Protein analysis confirmed a decrease of UT-B protein expression in bladder cancer and the level of UT-B protein is inversely associated with tumor malignancy. We also found abnormal sialylation of UT-B protein glycan in bladder cancer. Since the bladder is a unique organ holding high urea-containing urine, its urothelium is constantly in contact with a high concentration of urea. Aberrant (gene mutation) or absent expression of UT-B inevitably causes intracellular urea accumulation and subsequently may activate a carcinogenic pathway and/or enhance the bladder cell susceptibility to carcinogen exposure. The novel bladder cancer associated UT-B1Δ24 could be a new biomarker for bladder cancer diagnosis and prognosis. Future work is required to elucidate the mechanism about how UT-B gene expression switches from UT-B2 to UT-B1 and how the 24-nt deletion in UT-B1 occurs in bladder cancers.

### Data deposition

The sequence of the new UT-B1 gene has been deposited in Genbank with the accession number KY706499.

## Ethics statement

This study was carried out in accordance with the recommendations of the Ethics Committee of Emory University with written consent from all subjects. The protocol was approved by the Ethics Committee of Emory University (protocol number IRB: 00055316). All subjects gave written informed consent in accordance with the Declaration of Helsinki.

## Author contributions

GC, RH, MA, BY, JS, and XK conception, design, data analysis, and interpretation; RH and MA – sample and data collection; RH, and GC –experimental execution; GC, RH, MA, and JS – manuscript writing; GC – final approval of manuscript. All authors have read and approved the manuscript.

## Funding

This work was supported by Emory URC grant (to GC) and NIH grants R01-DK087838 (to GC), and R01-DK89828 and R01-DK41707 (to JS). RH was supported by the China Scholarship Council (CSC) under the State Scholarship Fund.

### Conflict of interest statement

The authors declare that the research was conducted in the absence of any commercial or financial relationships that could be construed as a potential conflict of interest.
